# Comparison of Cardiovascular Safety for Smoking Cessation Pharmacotherapies in a Population-Based Cohort in Australia

**DOI:** 10.1001/jamanetworkopen.2021.36372

**Published:** 2021-11-29

**Authors:** Alys Havard, Stephanie K. Y. Choi, Sallie-Anne Pearson, Clara K. Chow, Duong T. Tran, Kristian B. Filion

**Affiliations:** 1 National Drug and Alcohol Research Centre, University of New South Wales, Sydney, Sydney, New South Wales, Australia; 2 Centre for Big Data Research in Health, University of New South Wales, Sydney, Sydney, New South Wales, Australia; 3 Westmead Applied Research Centre, Faculty of Medicine and Health, The University of Sydney, Sydney, New South Wales, Australia; 4 Centre for Clinical Epidemiology, Lady Davis Research Institute, Jewish General Hospital, Montreal, Quebec, Canada; 5 Department of Medicine, McGill University, Montreal, Quebec, Canada; 6 Department of Epidemiology, Biostatistics, and Occupational Health, McGill University, Montreal, Quebec, Canada

## Abstract

**Importance:**

Although concerns exist regarding a potential increased risk of cardiovascular events for smoking cessation pharmacotherapies, there is general consensus that any increased risk associated with their use would be outweighed by the benefits of smoking cessation; thus, clinical guidelines recommend that such pharmacotherapies be offered to everyone who wants to quit smoking. In the interest of minimizing risk to patients, prescribers need evidence indicating how these pharmacotherapies compare with one another in terms of cardiovascular safety.

**Objective:**

To compare the risk of major adverse cardiovascular events (MACE) among individuals initiating varenicline, nicotine replacement therapy (NRT) patches, or bupropion.

**Design, Setting, and Participants:**

This retrospective, population-based cohort study using linked pharmaceutical dispensing, hospital admissions, and death data was conducted in New South Wales, Australia. Participants included adults who were dispensed a prescription smoking cessation pharmacotherapy between 2008 and 2015 or between 2011 and 2015, depending on the availability of the pharmacotherapies being compared. Pairwise comparisons were conducted for risk of MACE among 122 932 varenicline vs 92 148 NRT initiators; 342 064 varenicline vs 10 457 bupropion initiators; and 102 817 NRT vs 6056 bupropion initiators.

**Exposure:**

First course of the smoking cessation pharmacotherapy of interest.

**Main Outcomes and Measures:**

The primary outcome was MACE, defined as a composite of acute coronary syndrome, stroke, and cardiovascular death. Secondary outcomes were the individual components of MACE. Inverse probability of treatment weighting with high-dimensional propensity scores was used to account for potential confounding. Cox proportional hazards regression models with robust variance were used to estimate hazard ratios (HRs) and 95% CIs. Data were analyzed January 24, 2019, to September 1, 2021.

**Results:**

The mean (SD) age of included individuals ranged from 41.9 (14.2) to 49.8 (14.9) years, and the proportion of women ranged from 42.8% (52 702 of 123 128) to 52.2% (53 693 of 102 913). The comparison of 122 932 varenicline initiators and 92 148 NRT patch initiators showed no difference in the risk of MACE (HR, 0.87; 95% CI, 0.72-1.07) nor in the risk of the secondary outcomes of acute coronary syndrome (HR, 0.96; 95% CI, 0.76-1.21) and stroke (HR, 0.72; 95% CI, 0.45-1.14). However, decreased risk of cardiovascular death was found among varenicline initiators (HR, 0.49; 95% CI, 0.30-0.79). The results of comparisons involving bupropion were inconclusive owing to wide confidence intervals (eg, risk of MACE: 342 064 varenicline vs 10 457 bupropion initiators, HR, 0.87 [95% CI, 0.53-1.41]; 102 817 NRT patch vs 6056 bupropion initiators, HR, 0.79 [95% CI, 0.39-1.62]).

**Conclusions and Relevance:**

The finding of this cohort study that varenicline and NRT patch use have similar risk of MACE suggests that varenicline, the most efficacious smoking cessation pharmacotherapy, may be prescribed instead of NRT patches without increasing risk of major cardiovascular events. Further large-scale studies of the cardiovascular safety of varenicline and NRT relative to bupropion are needed.

## Key Points

**Question:**

What is the relative cardiovascular safety of the smoking cessation pharmacotherapies varenicline, nicotine replacement therapy (NRT) patches, and bupropion?

**Findings:**

In this cohort study of 342 064 varenicline and 10 457 bupropion initiators, 122 932 varenicline and 92 148 NRT patch initiators, and 102 817 NRT patch and 6056 bupropion initiators residing in New South Wales, Australia, no difference in the risk of major adverse cardiovascular events was found between the use of varenicline and NRT patches. The results of the comparisons for risk of major adverse cardiovascular events in the use of varenicline or NRT patches with the use of bupropion were inconclusive.

**Meaning:**

Findings from this cohort study suggest that varenicline, the most efficacious of the smoking cessation pharmacotherapies, may be prescribed in preference to NRT patches without increasing risk of major cardiovascular events.

## Teaser

This cohort study uses linked pharmaceutical dispensing, hospital admissions, and death data to compare the risk of major adverse cardiovascular events after initiation of the smoking cessation pharmacotherapies varenicline, nicotine replacement therapy patches, and bupropion among adults in New South Wales, Australia.

## Introduction

Smoking remains a leading preventable cause of morbidity and premature mortality, accounting for 6.4 million deaths worldwide in 2015.^1^ Quitting smoking substantially reduces the risk of developing cardiovascular disease, chronic obstructive pulmonary disease, and cancer, and it can extend life expectancy by up to 10 years.^2^

Clinical practice guidelines from most countries recommend that adults who want to quit smoking be offered smoking cessation pharmacotherapies.^3^ The efficacy of these medicines, which include bupropion, varenicline, and nicotine replacement therapy (NRT), is well established, with varenicline having the highest efficacy.^4^ For all 3 smoking cessation pharmacotherapies, concerns exist regarding possible adverse cardiovascular effects. These concerns were prompted by the nonstatistically significant increased risks of major adverse cardiovascular events (MACE) observed in some clinical trials and meta-analyses.^5,6,7,8,9,10^ Other meta-analyses have not found an increased risk.^4,11,12,13^ Because the pooled incidence is low even in those studies giving rise to concerns (≤1% in all treatment groups),^6,9^ there is a general consensus that any increased risk associated with the use of these pharmacotherapies would be small and outweighed by the benefits of smoking cessation.^14,15^ Nonetheless, in the interest of minimizing risk to patients, prescribers need evidence on how these medicines compare with each other in terms of cardiovascular safety.

Prior studies examining the risk of cardiovascular events among adults who used different smoking cessation pharmacotherapies generally measured outcomes for follow-up periods of 6 to 12 months.^6,16,17,18,19^ Follow-up periods of that length allow for the inclusion of outcomes occurring long after treatment completion or discontinuation, which may conflate the potential adverse effects of these medicines (ie, their safety) with the longer-term benefits of smoking cessation. Two studies examining the comparative safety of bupropion and varenicline avoided this problem by measuring outcomes only during treatment (ie, during medication coverage and the ensuing 7 days), with neither finding a difference.^20,21^ The objective of the present study was to examine the relative cardiovascular safety of all 3 smoking cessation pharmacotherapies by comparing the risk of MACE during treatment.

## Methods

This study followed the Strengthening the Reporting of Observational Studies in Epidemiology (STROBE) reporting guideline for cohort studies. This study was approved by the New South Wales (NSW) Population and Health Services Research Ethics Committee, the Aboriginal Health & Medical Research Council of NSW Ethics Committee, and the Australian Institute of Health and Welfare Ethics Committee. The NSW Population and Health Services Research Ethics Committee waived the requirement for obtaining informed consent in line with the NSW State Privacy Commissioner’s Guidelines for Research and the Health Records and Information Privacy Act 2002 and the Guidelines approved under Section 95/95A of the Australian Privacy Act 1988.

### Data Sources

This population-based cohort study used linked pharmaceutical dispensing, hospital, and death records. We obtained these data for all residents of NSW, Australia, who were dispensed a prescribed smoking cessation pharmacotherapy between July 1, 2002, and March 31, 2017. Australia has a publicly funded universal health care system with all eligible residents entitled to subsidized health services, including prescribed pharmaceuticals, through the Pharmaceutical Benefits Scheme (PBS). At the time of the study (2015), general beneficiaries paid a maximum of A$37.70 (equivalent to US $27.80) per dispensing, and social security recipients (referred to as concessional beneficiaries) paid A$6.10 (US $4.50).^22^

Pharmaceutical dispensing records were extracted from the PBS collection, which contains a record of every dispensed medicine for which a subsidy was paid. Since July 2012, the collection also includes records for PBS-listed medicines for which no subsidy was paid (ie, medicines that cost less than the copayment threshold). Hospital admission records were extracted from the NSW Admitted Patient Data Collection, which includes a record for every hospital separation from public and private hospitals in NSW. Diagnoses in those records are coded according to the *International Statistical Classification of Diseases and Related Health Problems, Tenth Revision, Australian Modification* (*ICD-10-AM*).^23^ The accuracy of this coding has been found to be high.^24^ Data on dates of death were obtained from the NSW Registry of Births, Deaths and Marriages, and cause-of-death data were extracted from the Australian Coordinating Registry Cause of Death Unit Record File. Causes are coded according to *ICD-10*, and at the time of extraction, these data were available only to December 31, 2015. The Centre for Health Record Linkage probabilistically linked the hospital and death records, and the Australian Institute of Health and Welfare performed the linkage to the PBS records.

### Smoking Cessation Pharmacotherapies

Bupropion and varenicline are medicines available by prescription only and have been listed with the PBS since February 2001 and January 2008, respectively. Prescription NRT patches have been subsidized for the entire Australian population since January 2011. Other forms of NRT (eg, gum, lozenges, and spray) were not listed with the PBS at the time of the present study. All formulations of NRT are also available over the counter, and these purchases are not captured in the PBS data. All 3 medicines are subsidized by the PBS only for the indication of smoking cessation, with annual limits on the amount available under subsidy (9 weeks for bupropion, 24 weeks for varenicline, and 12 weeks for NRT patches).

### Study Population

We created 3 study cohorts to conduct pairwise comparisons of the 3 pharmacotherapies, with study periods varying according to the availability of the included pharmacotherapies: varenicline vs bupropion (January 1, 2008, to December 31, 2015), varenicline vs prescription NRT patches, and prescription NRT patches vs bupropion (the latter 2 from January 1, 2011, to December 31, 2015). We included individuals in the cohort for a pairwise comparison if they initiated their first course of either pharmacotherapy during the corresponding study period. If an individual initiated both pharmacotherapies, we considered them exposed to the first dispensed pharmacotherapy only and censored follow-up on dispensing of the second pharmacotherapy. We used PBS records back to July 2002 to distinguish the first course from subsequent courses. The first recorded dispensing of the pharmacotherapy of interest during the study period was considered the index dispensing. We excluded anyone aged younger than 18 years at the index dispensing and individuals dispensed either of the other pharmacotherapies in the 6 months prior to their index dispensing.

### Exposure

In our main analysis, we defined exposure using an as-treated approach. We considered individuals exposed to the pharmacotherapy of interest from the date of index dispensing until discontinuation or switching to a different pharmacotherapy. Discontinuation was defined as the date when the amount dispensed would have been exhausted (estimated using the date of first dispensing, the quantity supplied, and the recommended daily dose as reported in the product information^25^) plus 30 days. In line with prior systematic reviews examining the cardiovascular safety of smoking cessation pharmacotherapies, we chose 30 days as a biologically relevant window for detecting adverse cardiovascular effects.^12,13^ Switching was defined as the dispensing of a different pharmacotherapy within the 30 days of the amount dispensed being exhausted. We observed participants until the first occurrence of the outcome or censoring due to discontinuation or switching, death from causes other than the outcome, or end of the study period (December 31, 2015, beyond which cause of death was not available), whichever occurred first.

### Outcomes

The primary outcome was the occurrence of MACE, defined as a composite of acute coronary syndrome (ACS) (*ICD-10-AM* codes I20.0 and I21.x-I22.x), stroke (*ICD-10-AM* codes I60.x, I61.x, I63.x, and I64.x), and cardiovascular death (*ICD-10* codes I00.x-I99.x and R96.x). Secondary outcomes were the individual components of MACE. We identified ACS and stroke from both hospital and death records and cardiovascular death from death records alone. We searched only the primary diagnosis field in hospital data and the underlying cause of death field in death data.

### Potential Confounders

Potential confounders included the following sociodemographic characteristics ascertained from the index dispensing record: age, sex, calendar year, type of PBS beneficiary, socioeconomic status of residential area (based on the Index of Relative Socio-economic Disadvantage^26^), and geographic remoteness of residential area (based on the Australian Statistical Geography Standard^27^). Preexisting cardiovascular disease, other morbidities, and use of medicines known to be associated with cardiovascular outcomes and plausibly related to treatment choice (eTable 1 in the Supplement) were identified from dispensing records and hospital-recorded diagnoses in the 5 years prior to the index dispensing.

### Statistical Analysis

To account for potential confounding, we used inverse probability of treatment weighting^28^ with high-dimensional propensity scores.^29^ For each outcome in each pairwise comparison, we used logistic regression to construct a propensity model that included the prespecified potential confounders described and 500 empirically identified covariates. We generated stabilized weights to minimize the effect of extreme weights^30^ and then trimmed individuals with weights of 10 or higher.^31^ We also used graphical methods to compare the cumulative distribution of the propensity scores before and after weighting.^28^ We calculated standardized differences to assess balance in the characteristics of the weighted treatment groups, with differences in their absolute values less than 0.1 considered negligible.^30^

For each outcome, we calculated incidence rates in each of the weighted treatment groups, with 95% CIs estimated with the jackknife method.^32^ We also constructed weighted adjusted survival curves for all outcomes and fitted Cox proportional hazards regression models with robust variance to estimate hazard ratios (HRs) and 95% CIs.^33^ We do not report HRs when there were fewer than 5 events in either exposure group.^34^ The proportionality assumption of each model was examined using martingale-based residuals.^35^

For the primary outcome only, we conducted a subgroup analysis focused on individuals with preexisting cardiovascular disease, defined as individuals with a hospital admission in the 5 years prior to the index dispensing in which the diagnosis (primary and secondary) or procedure fields contained 1 or more codes listed in eTable 2 in the Supplement. Given the potential for bias from informative censoring in as-treated analyses, we conducted sensitivity analyses using an approach that is analogous to an intention-to-treat approach. In this analysis, we followed participants until the occurrence of the outcome, censoring due to death from causes other than the outcome, end of the study period (December 31, 2015), or a maximum follow-up of 6 months, whichever occurred first. We did not censor individuals on pharmacotherapy discontinuation or switching. In a post hoc sensitivity analysis testing the robustness of our measurement of cardiovascular death, we included all-cause mortality as a secondary outcome. Data were analyzed January 24, 2019, to September 1, 2021, using Stata, version 16 (StataCorp LLC)

## Results

### Study Cohorts

Application of our inclusion criteria (Figure), followed by removal of individuals with extreme weights, resulted in the following cohort sizes for our analysis of MACE: 342 064 varenicline initiators and 10 457 bupropion initiators; 122 932 varenicline initiators and 92 148 NRT patch initiators; and 102 817 NRT patch initiators and 6056 bupropion initiators. The sizes of the final cohorts for the secondary outcomes were similar.

**Figure.**
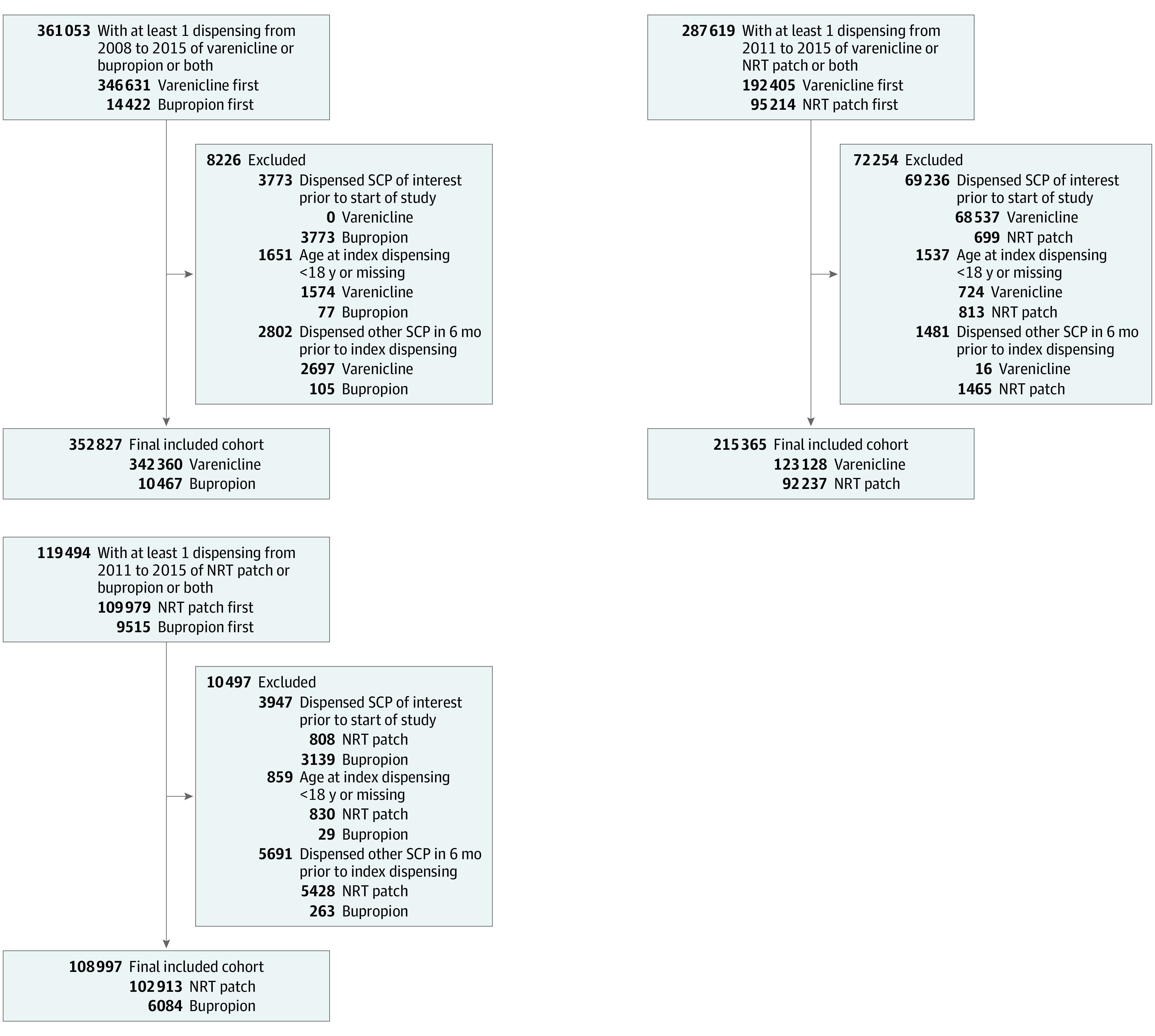
Cohort Selection Diagram NRT indicates nicotine replacement therapy; SCP, smoking cessation pharmacotherapy.

The median number of tablets dispensed to varenicline initiators was 53 (IQR, 53-165) and 30 (IQR, 30-120) tablets for bupropion initiators. The NRT patch initiators were dispensed a median of 28 patches (IQR, 28-56 patches), 92% of which were the highest strength available (21-25 mg of nicotine per day). The median follow-up time was 58 days (IQR, 58-142 days) for varenicline initiators in both cohorts and 58 days (IQR, 58-144 days) for both sets of NRT patch initiators. The median follow-up time was 62 days for both sets of bupropion initiators (IQR, 62-123 days when compared with varenicline; IQR, 62-124 days when compared with NRT patches).

### Cohort Characteristics

The mean (SD) age across treatment groups ranged from 41.9 (14.2) to 49.8 (14.9) years, and the proportion of women ranged from 42.8% (52 702 of 123 128) to 52.2% (53 693 of 102 913), whereas the proportion of men ranged from 47.8% (49 220 of 102 913) to 57.2% (70 426 of 123 128). Prior to weighting, varenicline and bupropion initiators were similar with respect to most baseline characteristics (eFigure in the Supplement) except varenicline initiators were less likely to have their index dispensing in 2008 but more likely to have it in 2009, 2010, and 2011 (Table 1 and eTables 3, 4, and 5 in the Supplement). Varenicline initiators were also less likely to live in the least socioeconomically disadvantaged areas and major cities and were less likely to have a history of psychiatric conditions.

**Table 1.** Baseline Characteristics of Smoking Cessation Pharmacotherapy Initiators Included in the Analysis of MACE, for Each Pairwise Comparison^a^

Characteristic^b^	Varenicline vs bupropion				Varenicline vs NRT patch				NRT patch vs bupropion			
	No. (%) of group		Standardized difference		No. (%) of group		Standardized difference		No. (%) of group		Standardized difference	
	Varenicline (n = 342 360)	Bupropion (n = 10 467)	Before weighting	After weighting	Varenicline (n = 123 128)	NRT (n = 92 237)	Before weighting	After weighting	NRT (n = 102 913)	Bupropion (n = 6084)	Before weighting	After weighting
Age, mean (SD), y	43.7 (14.1)	43.6 (14.2)	0.066	0.046	41.9 (14.2)	49.8 (14.9)	0.548	0.002	49.8 (14.9)	42.9 (13.6)	0.486	0.028
Women	157 762 (46.1)	5040 (48.1)	0.041	0.013	52 702 (42.8)	48 073 (52.1)	0.187	0.004	53 693 (52.2)	3043 (50.0)	0.043	0.022
Men	184 598 (53.9)	5427 (51.8)	0.041	0.013	70 426 (57.2)	44 164 (47.9)	0.187	0.004	49 220 (47.8)	3041 (50.0)	0.043	0.022
Beneficiary category												
General	196 481 (57.4)	5764 (55.1)	0.047	0.036	73 441 (59.6)	26 074 (28.3)	0.666	0.008	29 408 (28.6)	3432 (56.4)	0.587	0.018
Concessional	144 008 (42.1)	4630 (44.2)	0.044	0.037	49 261 (40.0)	65 439 (70.9)	0.655	0.021	72 711 (70.7)	2615 (43.0)	0.582	0.015
Veterans	1872 (0.5)	73 (0.7)	0.019	0.002	426 (0.3)	724 (0.8)	0.058	0.075	794 (0.8)	37 (0.6)	0.020	0.124
Socioeconomic status quintile												
1 (Most disadvantaged)	63 406 (18.5)	1806 (17.3)	0.033	0.061	24 916 (20.2)	21 074 (22.8)	0.064	0.009	23 647 (23.0)	1080 (17.8)	0.130	0.049
2	77 125 (22.5)	2109 (20.1)	0.058	0.007	25 785 (20.9)	20 848 (22.6)	0.040	0.017	23 323 (22.7)	1206 (19.8)	0.069	0.029
3	91 161 (26.6)	2660 (25.4)	0.028	0.041	31 887 (25.9)	23 450 (25.4)	0.011	0.006	26 365 (25.6)	1563 (25.7)	0.001	0.028
4	65 347 (19.1)	1912 (18.3)	0.021	0.079	23 251 (18.9)	16 479 (17.9)	0.026	0.008	18 278 (17.8)	1147 (18.9)	0.028	0.071
5 (Least disadvantaged)	45 321 (13.2)	1980 (18.9)	0.155	0.076	17 290 (14.0)	10 386 (11.3)	0.084	0.012	11 301 (11.0)	1088 (17.9)	0.197	0.016
Remoteness of residence												
Major cities	174 040 (50.8)	5860 (56.0)	0.103	0.018	66 313 (53.9)	48 855 (53.0)	0.018	0.018	54 016 (52.5)	3232 (53.1)	0.013	0.040
Inner regional	86 691 (25.3)	2432 (23.2)	0.049	0.044	28 478 (23.1)	23 844 (25.9)	0.063	0.052	26 739 (26.0)	1482 (24.4)	0.037	0.057
Outer regional	72 672 (21.2)	1888 (18.0)	0.080	0.009	25 651 (20.8)	17 745 (19.2)	0.040	0.033	20 111 (19.5)	1196 (19.7)	0.003	0.004
Remote	5426 (1.6)	228 (2.2)	0.044	0.096	1547 (1.3)	1033 (1.1)	0.013	0.008	1186 (1.2)	116 (1.9)	0.062	0.063
Very remote	3531 (1.0)	58 (0.6)	0.054	0.011	1138 (0.9)	760 (0.8)	0.011	0.005	861 (0.8)	57 (0.9)	0.011	0.013
Index prescription, y												
2008	77 875 (22.7)	4529 (43.3)	0.447	0.017	NA	NA	NA	NA	NA	NA	NA	NA
2009	76 028 (22.2)	1561 (14.9)	0.188	0.002	NA	NA	NA	NA	NA	NA	NA	NA
2010	61 587 (18.0)	1041 (9.9)	0.234	0.003	NA	NA	NA	NA	NA	NA	NA	NA
2011	39 762 (11.6)	721 (6.9)	0.164	0.004	40 330 (32.8)	41 741 (45.3)	0.258	0.002	42 164 (41.0)	1305 (21.5)	0.431	0.002
2012	27 427 (8.0)	695 (6.6)	0.053	0.005	26 805 (21.8)	18 086 (19.6)	0.053	0.001	20 264 (19.7)	1189 (19.5)	0.004	0.022
2013	22 642 (6.6)	646 (6.2)	0.018	0.010	21 522 (17.5)	12 457 (13.5)	0.110	0.000	15 008 (14.6)	1215 (20.0)	0.143	0.010
2014	19 796 (5.8)	655 (6.3)	0.020	0.000	18 510 (15.0)	10 202 (11.1)	0.118	0.002	12 772 (12.4)	1184 (19.5)	0.194	0.002
2015	17 243 (5.0)	619 (5.9)	0.039	0.001	15 961 (13.0)	9750 (10.6)	0.074	0.001	12 705 (12.3)	1190 (19.6)	0.198	0.011
Morbidities and medicine use												
Gastroesophageal reflux	84 805 (24.8)	2657 (25.4)	0.014	0.027	26 810 (21.8)	33 978 (36.8)	0.336	0.005	38 297 (37.2)	1594 (26.2)	0.238	0.017
Diabetes	20 665 (6.0)	638 (6.1)	0.003	0.003	7005 (5.7)	10 174 (11.0)	0.194	0.001	11 577 (11.2)	395 (6.5)	0.168	0.009
Blood disorder	32 110 (9.4)	962 (9.2)	0.007	0.012	9318 (7.6)	16 588 (18.0)	0.316	0.012	18 564 (18.0)	537 (8.8)	0.273	0.021
Arrhythmia	3427 (1.0)	104 (1.0)	0.001	0.013	898 (0.7)	1938 (2.1)	0.116	0.004	2138 (2.1)	64 (1.1)	0.083	0.043
Hypertension	36 293 (10.6)	1090 (10.4)	0.006	0.023	11 466 (9.3)	18 925 (20.5)	0.319	0.008	21 311 (20.7)	731 (12.0)	0.237	0.000
Hyperlipidemia	59 731 (17.4)	1701 (16.3)	0.032	0.041	17 882 (14.5)	26 392 (28.6)	0.348	0.006	29 724 (28.9)	1018 (16.7)	0.293	0.030
Oral corticosteroid	38 705 (11.3)	1128 (10.8)	0.017	0.033	13 637 (11.1)	19 222 (20.8)	0.269	0.009	22 106 (21.5)	880 (14.5)	0.184	0.050
Thyroid disease	9192 (2.7)	339 (3.2)	0.033	0.012	2999 (2.4)	4776 (5.2)	0.144	0.002	5374 (5.2)	242 (4.0)	0.059	0.049
Malignant neoplasm	4477 (1.3)	144 (1.4)	0.006	0.010	1447 (1.2)	2342 (2.5)	0.101	0.004	2629 (2.6)	78 (1.3)	0.093	0.021
NSAIDs	87 578 (25.6)	2845 (27.2)	0.036	0.035	27 067 (22.0)	32 736 (35.5)	0.302	0.005	37 240 (36.2)	1622 (26.7)	0.206	0.015
Epilepsy	15 814 (4.6)	697 (6.7)	0.088	0.005	4646 (3.8)	9248 (10.0)	0.249	0.012	10 158 (9.9)	446 (7.3)	0.090	0.034
Psychotic illness	17 065 (5.0)	1067 (10.2)	0.198	0.031	6323 (5.1)	14 270 (15.5)	0.345	0.024	15 561 (15.1)	748 (12.3)	0.082	0.023
Anxiety	42 253 (12.3)	1688 (16.1)	0.109	0.028	13 465 (10.9)	24 297 (26.3)	0.404	0.018	27 001 (26.2)	1181 (19.4)	0.163	0.007
Mood disorder	97 149 (28.4)	3824 (36.5)	0.175	0.040	32 010 (26.0)	43 894 (47.6)	0.459	0.006	49 104 (47.7)	2574 (42.3)	0.109	0.001
Alcohol or drug dependence	21 314 (6.2)	918 (8.8)	0.097	0.015	7321 (5.9)	13 179 (14.3)	0.279	0.021	14 376 (14.0)	575 (9.4)	0.141	0.011
Chronic airway disease	92 486 (27.0)	2879 (27.5)	0.011	0.022	29 996 (24.4)	37 647 (40.8)	0.357	0.008	42 658 (41.5)	1780 (29.3)	0.257	0.023
Kidney disease	3141 (0.9)	88 (0.8)	0.008	0.006	918 (0.7)	1736 (1.9)	0.100	0.010	1919 (1.9)	59 (1.0)	0.076	0.015
Rheumatic diseases	534 (0.2)	18 (0.2)	0.004	0.021	109 (0.1)	292 (0.3)	0.051	0.008	312 (0.3)	5 (0.1)	0.050	0.011
Heart failure and cardiomyopathy	797 (0.2)	19 (0.2)	0.011	0.008	222 (0.2)	650 (0.7)	0.079	0.004	724 (0.7)	16 (0.3)	0.063	0.039
Acute coronary syndrome	6210 (1.8)	163 (1.6)	0.020	0.028	1602 (1.3)	3502 (3.8)	0.159	0.023	3891 (3.8)	81 (1.3)	0.156	0.019
Other ischemic heart disease	8427 (2.5)	246 (2.4)	0.007	0.012	2042 (1.7)	4349 (4.7)	0.175	0.014	4853 (4.7)	119 (2.0)	0.154	0.007
Cerebrovascular disease	3155 (0.9)	94 (0.9)	0.002	0.005	848 (0.7)	2161 (2.3)	0.136	0.009	2424 (2.4)	53 (0.9)	0.118	0.088
Peripheral arterial disease	1274 (0.4)	32 (0.3)	0.011	0.003	372 (0.3)	771 (0.8)	0.071	0.005	877 (0.9)	25 (0.4)	0.056	0.062
Percutaneous coronary interventions	1131 (0.3)	29 (0.3)	0.010	0.001	278 (0.2)	569 (0.6)	0.060	0.003	635 (0.6)	18 (0.3)	0.048	0.026
Coronary artery bypass grafting	843 (0.2)	25 (0.2)	0.001	0.001	208 (0.2)	480 (0.5)	0.060	0.002	531 (0.5)	13 (0.2)	0.050	0.026

Abbreviations: MACE, major adverse cardiovascular events; NA, not applicable; NRT, nicotine replacement therapy; NSAIDs, nonsteroidal anti-inflammatory drug.

^a^ These characteristics are for participants included in the analysis of MACE. Owing to slight differences in the cohort selection criteria and modest differences in the high-dimensional propensity score distributions, minor differences exist between the characteristics of participants included in the analysis of MACE and participants included in the analysis of other outcomes (eTables 3, 4, and 5 in the Supplement).

^b^ Less than 0.1% of participants had missing data for these characteristics; such participants were removed from the analysis owing to the inability to compute their propensity score.

By contrast, there were several differences between initiators of varenicline and initiators of NRT patches and between initiators of an NRT patch and initiators of bupropion (Table 1; eTables 3, 4, and 5 and the eFigure in the Supplement). The NRT patch initiators were older and more likely to have their index dispensing early in the study period compared with both varenicline and bupropion initiators. The NRT patch initiators were more likely than varenicline initiators to be women. The NRT patch initiators were also more likely to be concessional beneficiaries, and when compared with bupropion initiators, they were more likely to live in the most socioeconomically disadvantaged areas. The NRT patch initiators were more likely to have preexisting cardiovascular disease and other morbidities and to use medicines known to be associated with cardiovascular outcomes compared with both varenicline and bupropion initiators.

After weighting, we did not observe meaningful differences in baseline characteristics except in our analyses of MACE, stroke, and cardiovascular death, with NRT patch initiators being more likely than bupropion initiators to be veterans (Table 1; eTable 5 in the Supplement). We adjusted for these differences.

### Cardiovascular Safety

The overall incidence rate for MACE among varenicline initiators and NRT patch initiators was 11.77 per 1000 person-years (95% CI, 10.63-13.07 per 1000 person-years), with no between-group differences in the risk of MACE (HR, 0.87; 95% CI, 0.72-1.07) or the secondary outcomes of ACS (HR, 0.96; 95% CI, 0.76-1.21) and stroke (HR, 0.72; 95% CI, 0.45-1.14). However, varenicline was associated with a decreased risk of cardiovascular death (HR, 0.49; 95% CI, 0.30-0.79). In absolute terms, varenicline was associated with 1.5 fewer cardiovascular deaths per 1000 person-years of exposure relative to NRT patches (Table 2). The sensitivity analysis using an intention-to-treat approach yielded similar results for MACE, ACS, and stroke, and although the results for cardiovascular death were attenuated, the association persisted (HR, 0.67; 95% CI, 0.47-0.95) (eTable 6 in the Supplement). In the subgroup analysis focused on patients with preexisting cardiovascular disease, we again found no difference in the risk of MACE, although the 95% CI was somewhat wide (HR, 0.77; 95% CI, 0.54-1.12) (eTable 7 in the Supplement). Our sensitivity analysis with all-cause death as the outcome yielded a similar result to that for cardiovascular death (HR, 0.31; 95% CI, 0.23-0.41) (eTable 8 in the Supplement).

**Table 2.** Hazard Ratios for Cardiovascular Outcomes Associated With Smoking Cessation Pharmacotherapy Initiation, for Each Pairwise Comparison^a^

Exposure	No. of individuals^b^	No. of events	No. of person-years	Incidence rate, per 1000 person-years (95% CI)	Hazard ratio (95% CI)
MACE					
Varenicline	342 064	751	87 881	8.54 (7.96-9.18)	0.87 (0.53-1.41)
Bupropion	10 457	26	2578	9.94 (6.19-17.02)	1 [Reference]
ACS					
Varenicline	342 064	592	87 880	6.74 (6.22-7.31)	0.91 (0.57-1.45)
Bupropion	10 458	19	2582	7.50 (4.78-12.45)	1 [Reference]
Stroke					
Varenicline	324 064	118	87 881	1.35 (1.13-1.62)	Not reported^d^
Bupropion	10 457	<5	Suppressed^c^	1.04 (0.11-43.81)	
CV death					
Varenicline	342 064	97	87 883	1.10 (0.90-1.35)	0.50 (0.14-1.77)
Bupropion	10 457	6	2579	2.28 (0.54-18.33)	1 [Reference]
MACE					
Varenicline	122 932	356	32 304	11.03 (9.41-13.03)	0.87 (0.72-1.07)
NRT	92 148	269	20 857	12.92 (11.62-14.40)	1 [Reference]
ACS					
Varenicline	122 927	268	32 307	8.30 (6.90-10.06)	0.96 (0.76-1.21)
NRT	92 148	186	20 854	8.91 (7.81-10.23)	1 [Reference]
Stroke					
Varenicline	122 937	60	32 310	1.84 (1.23-2.91)	0.72 (0.45-1.14)
NRT	92 148	53	20 864	2.53 (2.04-3.19)	1 [Reference]
CV death					
Varenicline	122 930	45	32 329	1.39 (0.92-2.22)	0.49 (0.30-0.79)
NRT	92 148	61	20 851	2.91 (2.41-3.55)	1 [Reference]
MACE					
NRT	102 817	423	24 409	17.34 (15.80-19.07)	0.79 (0.39-1.62)
Bupropion	6056	32	1447	22.28 (10.89-53.03)	1 [Reference]
ACS					
NRT	102 817	272	24 410	11.16 (9.94-12.58)	0.74 (0.34-1.62)
Bupropion	6049	21	1426	14.95 (6.88-38.83)	1 [Reference]
Stroke					
NRT	103 636	95	24 566	3.85 (3.17-4.73)	Not reported^d^
Bupropion	6086	<5	Suppressed^c^	3.03 (0.58-38.09)	
CV death					
NRT	102 817	110	24 415	4.50 (3.76-5.43)	Not reported^d^
Bupropion	6049	<5	Suppressed^c^	3.06 (0.50-52.02)	

Abbreviations: ACS, acute coronary syndrome; CV, cardiovascular; MACE, major adverse cardiovascular events; NRT, nicotine replacement therapy (patch).

^a^ Main analyses using an as-treated approach. Treatment groups were weighted using inverse probability of treatment weighting with high-dimensional propensity scores.

^b^ Varies across comparisons owing to removal of individuals with weights of 10 or higher.

^c^ Cell value suppressed because it was based on fewer than 5 individuals.

^d^ Hazard ratio not reported owing to fewer than 5 events in at least 1 of the exposure groups.

The overall incidence rate for MACE was 8.58 per 1000 person-years (95% CI, 8.00-9.22 per 1000 person-years) in varenicline and bupropion initiators and 17.62 per 1000 person-years (95% CI, 15.95-19.51 per 1000 person-years) in NRT patch and bupropion initiators. The results of our comparisons involving bupropion were inconclusive owing to wide 95% CIs around the HRs and, in some cases, an inability to estimate HRs owing to sparse data. Although the HR point estimates do not indicate large differences in the risk of MACE between varenicline and bupropion initiators (HR, 0.87; 95% CI, 0.53-1.41) or NRT patch and bupropion initiators (HR, 0.79; 95% CI, 0.39-1.62), the wide CIs mean that we cannot rule out clinically important differences in their risk. By contrast, results were suggestive of a decreased risk of cardiovascular death among varenicline initiators relative to bupropion initiators (HR, 0.50; 95% CI, 0.14-1.77), but again not conclusive. Our intention-to-treat sensitivity analysis also yielded wide 95% CIs and inconclusive results (eTable 6 in the Supplement), and our subgroup analysis, which focused on patients with preexisting cardiovascular disease, was uninformative owing to sparse data (eTable 7 in the Supplement). Consistent with our analysis of cardiovascular death, our all-cause death analysis showed a decreased risk of death among varenicline initiators relative to bupropion initiators (HR, 0.43; 95% CI, 0.24-0.76). We also found an increased risk of death among NRT patch initiators relative to bupropion initiators, although the 95% CI was wide (HR, 2.39; 95% CI, 1.03-5.52) (eTable 8 in the Supplement).

## Discussion

In this population-based cohort study, we found no difference between varenicline and NRT patch use in the risk of MACE, ACS, or stroke. By contrast, we found a decreased risk of cardiovascular death among varenicline initiators, albeit small in absolute magnitude (1.5 fewer cardiovascular deaths per 1000 person-years). Two prior studies comparing the risk of major cardiovascular events among adults using varenicline and NRT found a lower risk of some outcomes among varenicline users. However, because these outcomes were measured for follow-up periods of 6 to 12 months^17,18^ (ie, follow-up durations that exceed the typical duration of use of smoking pharmacotherapies), it is unclear whether these lower risks were indicative of greater cardiovascular safety or due to potentially higher rates of smoking cessation in the varenicline group. This point raises the question of whether the lower risk of cardiovascular death among the varenicline initiators in our study might also be due to greater smoking cessation in this group. We consider this option unlikely given that the median follow-up time was 58 days, and it takes 1 to 3 years of smoking abstinence to halve cardiovascular risk.^2,36^

This finding that varenicline use is similar to NRT patch use in terms of risk of MACE—and may be protective against some cardiovascular outcomes—is encouraging. Together with evidence that varenicline is the most efficacious smoking cessation pharmacotherapy,^4^ these findings suggest that varenicline may be prescribed in preference to NRT patches without fear of increasing the risk of major cardiovascular events. Such prescribing should have a downstream effect of increased smoking cessation and reduced cardiovascular disease burden among former smokers. However, this conclusion may not apply to individuals with preexisting cardiovascular disease; our subgroup analyses were uninformative owing to sparse data. Previously, preferential prescribing of varenicline may have raised concerns about potential neuropsychiatric symptoms (eg, suicidality and aggression), but these concerns have been allayed by mounting evidence^4,37,38,39^ and the lifting of the requirement for a boxed label warning regarding psychiatric adverse effects.^40^

The results of our comparisons involving bupropion were inconclusive but were suggestive of a benefit of varenicline over bupropion with respect to risk of cardiovascular death. Although prior studies of the comparative safety of varenicline and bupropion did not measure cardiovascular death,^20,21^ a study examining the risk of all-cause death found a decreased risk among elderly patients using varenicline.^20^ Together, these findings indicate that further exploration of the relative safety of varenicline and bupropion is warranted. The same applies to the relative safety of NRT patches and bupropion because our analysis of all-cause death showed a greater risk among patients using NRT patches (HR, 2.39; 95% CI, 1.03-5.52). Given the wide 95% CI and post hoc nature of this sensitivity analysis, this finding should be interpreted with caution.

### Limitations

Despite our use of sophisticated methods to control for a comprehensive range of potential confounders, we acknowledge the risk of residual confounding from unmeasured factors, with heaviness of smoking being a noteworthy example. In addition, we had no information about the actual use of medicines or the duration of use, in which nonuse of these medicines would have led to an underestimate of the risk of adverse effects. In addition, our study was limited to prescription NRT subsidized by the Australian government (only patches at the time of the study). This data limitation could have led to some misclassification, with varenicline and bupropion users potentially using over-the-counter NRT simultaneously and subsidized NRT patch users potentially supplementing with additional over-the-counter NRT products. This possibility may mean that we have overestimated the risk of harm associated with single use of any of these pharmacotherapies. One might hypothesize that this overestimation has occurred to a greater extent for NRT patch initiators; combination NRT is recommended in Australian guidelines^3^ and is therefore likely to be the most popular of these potential combinations. Finally, there may have been some outcome misclassification, with previous research reporting that 1.9% of admissions to Australian hospitals are for patients from other states.^41^

## Conclusions

The finding of this cohort study that varenicline and NRT patch use have similar risk of MACE suggests that varenicline, the most efficacious smoking cessation pharmacotherapy, may be prescribed instead of NRT patches without increasing risk of major cardiovascular events. Further large-scale studies of the cardiovascular safety of varenicline and NRT relative to bupropion are needed.

## Notes

Supplement. **eTable 1.** Identification of the Prespecified Potential Confounders **eTable 2.** Diagnosis and Procedure Codes Used to Identify Patients With a History of Cardiovascular Disease **eTable 3.** Baseline Characteristics of Smoking Cessation Pharmacotherapy Initiators in the Comparison of Varenicline and Bupropion, for Analyses of ACS, Stroke and Cardiovascular Death **eTable 4.** Baseline Characteristics of Smoking Cessation Pharmacotherapy Initiators in the Comparison of Varenicline and NRT Patch, for Analyses of ACS, Stroke and Cardiovascular Death **eTable 5.** Baseline Characteristics of Smoking Cessation Pharmacotherapy Initiators in the Comparison of NRT Patch and Bupropion, for Analyses of ACS, Stroke and Cardiovascular Death **eTable 6.** Hazard Ratios for Cardiovascular Outcomes Associated With Smoking Cessation Pharmacotherapy Initiation, for Each Pairwise Comparison **eTable 7.** Hazard Ratios for MACE Associated With Smoking Cessation Pharmacotherapy Initiation, Among Patients With a History of Cardiovascular Disease **eTable 8.** Hazard Ratios for All-Cause Death Outcome Associated With Smoking Cessation Pharmacotherapy Initiation, for Each Pairwise Comparison **eFigure.** High-Dimensional Propensity Score Distribution Among Smoking Cessation Pharmacotherapy Initiators Before and After Weighting
