# Supplementary material for: Comparison of Cardiovascular Safety for Smoking Cessation Pharmacotherapies in a Population-Based Cohort in Australia
Source: JAMA Netw Open. 2021 Nov 29;4(11):e2136372. doi: 10.1001/jamanetworkopen.2021.36372 (PMC8630569; doi:10.1001/jamanetworkopen.2021.36372)
Supplement: Supplement. — eTable 1. Identification of the Prespecified Potential Confounders eTable 2. Diagnosis and Procedure Codes Used to Identify Patients With a History of Cardiovascular Disease eTable 3. Baseline Characteristics of Smoking Cessation Pharmacotherapy Initiators in the Comparison of Varenicline and Bupropion, for Analyses of ACS, Stroke and Cardiovascular Death eTable 4. Baseline Characteristics of Smoking Cessation Pharmacotherapy Initiators in the Comparison of Varenicline and NRT Patch, for Analyses of ACS, Stroke and Cardiovascular Death eTable 5. Baseline Characteristics of Smoking Cessation Pharmacotherapy Initiators in the Comparison of NRT Patch and Bupropion, for Analyses of ACS, Stroke and Cardiovascular Death eTable 6. Hazard Ratios for Cardiovascular Outcomes Associated With Smoking Cessation Pharmacotherapy Initiation, for Each Pairwise Comparison eTable 7. Hazard Ratios for MACE Associated With Smoking Cessation Pharmacotherapy Initiation, Among Patients With a History of Cardiovascular Disease eTable 8. Hazard Ratios for All-Cause Death Outcome Associated With Smoking Cessation Pharmacotherapy Initiation, for Each Pairwise Comparison eFigure. High-Dimensional Propensity Score Distribution Among Smoking Cessation Pharmacotherapy Initiators Before and After Weighting [file jamanetwopen-e2136372-s001.pdf]

Supplementary Online Content

Havard A, Choi SKY, Pearson SA, Chow CK, Tran DT, Filion KB. Comparison of cardiovascular safety for smoking cessation pharmacotherapies in a population-based cohort in Australia. *JAMA Netw Open*. 2021;4(11):e2136372. doi:10.1001/jamanetworkopen.2021.36372

eTable 1. Identification of the Prespecified Potential Confounders

eTable 2. Diagnosis and Procedure Codes Used to Identify Patients With a History of Cardiovascular Disease

eTable 3. Baseline Characteristics of Smoking Cessation Pharmacotherapy Initiators in the Comparison of Varenicline and Bupropion, for Analyses of ACS, Stroke and Cardiovascular Death

eTable 4. Baseline Characteristics of Smoking Cessation Pharmacotherapy Initiators in the Comparison of Varenicline and NRT Patch, for Analyses of ACS, Stroke and Cardiovascular Death

eTable 5. Baseline Characteristics of Smoking Cessation Pharmacotherapy Initiators in the Comparison of NRT Patch and Bupropion, for Analyses of ACS, Stroke and Cardiovascular Death

eTable 6. Hazard Ratios for Cardiovascular Outcomes Associated With Smoking Cessation Pharmacotherapy Initiation, for Each Pairwise Comparison

eTable 7. Hazard Ratios for MACE Associated With Smoking Cessation Pharmacotherapy Initiation, Among Patients With a History of Cardiovascular Disease

eTable 8. Hazard Ratios for All-Cause Death Outcome Associated With Smoking Cessation Pharmacotherapy Initiation, for Each Pairwise Comparison

eFigure. High-Dimensional Propensity Score Distribution Among Smoking Cessation Pharmacotherapy Initiators Before and After Weighting

This supplementary material has been provided by the authors to give readers additional information about their work.

eTable 1. Identification of the Prespecified Potential Confounders

Characteristics	Data Source	Categories
Age at index dispensing date	PBS	Continuous variable
Sex	PBS	Women, men
Beneficiary category	PBS	Concessional patients, general patients, veterans
Socioeconomic status for areas	PBS	Quintile 1 to 5,<br>1= most disadvantaged<br>5= least disadvantaged
Remoteness of residence	PBS	Major cities, inner regional, outer regional, remote, very remote
Index prescription, year	PBS	2008-2015
**History of major cardiovascular diseases**	**Data Sources**	**Diagnosis & procedure codes**
See eTable 2	APDC	See eTable 2
**History of morbidities and medicine use**	**ATC Codes for identifying medicine use**	**Diagnosis Codes**
Gastroesophageal reflux disease	A02BA01-A02BX05	K21.0, K21.9
Diabetes	A10AA01-A10BX99, A10AD	E10, E11, E13
Blood disorder	Anticoagulants<br>B01AA03-B01AB06, B01AE07, B01AF01, B01AF02, B01AX05<br><br>Antiplatelets<br>B01AC04-B01AC07,<br>B01AC12-B01AC30, and following items: 05030R, 05035B, 05042J, 10111E, 10117L, 10129D, 10130E, 05751Q, 06456T.	D56, D57, D50-D53, D55, D58-D64, D65-D68
Arrhythmia	C01AA05, C01BA01-C01BD01, C07AA07	I45.6-I459, I46.0 I46.9, I47, I48, I49
Hypertension	C03AA01–C03BA11, C03DB01, C03DB99, C03EA01, C09BA02–C09BA09, C09DA02–C09DA08, C02AB01–C02AC05, C02DB02–C02DB99 (C03CA01–C03CCO1 or C09CA01–C09CX99)<br>C07AA01–C07AA06, C07AA08–C07AB01, C07AB02—if PBS item code is not 8732N, 8733P, 8734Q, 8735R, C07AG01, C08CA01–C08DB01, C09DB01–C09DB04, C09DX01, C09BB02–C09BB10, C07AB03, C09DX03, C10BX03	
Hyperlipidaemia	A10BH03, C10AA01–C10BX09	

© 2021 Havard A et al. *JAMA Network Open*.

History of morbidities and medicine use	ATC Codes for identifying medicine use	Diagnosis Codes
Oral corticosteroid	H02AB01-H02AB10	
Thyroid disease	H03BA02, H03BB01, H03AA01-H03AA02	E00-E07, E89.0
Malignant neoplasm	L01AA01-L01XX41	C31-C34, C39, C00-C15
NSAIDs	M01AB01-M01AH06	
Epilepsy	N03AA01-N03AX99	G40, F80.3
Psychotic illness	N05AA01-N05AB02, N05AB06-N05AL07, N05AX07-N05AX13	F20-25, F28-30
Anxiety	N05BA01-N05BA12, N05BE01	F40, F41, F44, F48
Mood disorder (bipolar disorder & depression)	N05AN01, N06AA01-N06AG02, N06AX03-N06AX11, N06AX13-N06AX18, N06AX21-N06AX26	F31-34. F38-39
Alcohol and drug dependency	N07BB01-N07BB99	F10-F16, F18-19 Z50.2, Z72.1, Z50.3, Z72.2
Chronic airways disease	R03AC02-R03DC03, R03DX05	J31, J32, J35, J37, J40-47, J98-99, R05
Kidney disease	A11CC01-A11CC04, B03XA01-B03XA03, V03AE02, V03AE03, V03AE05	N00-08, N11-12, N14-19, N25-28, N39.1, N39.2, Q60-Q63, T82.4, T86.1, Z49, Z94.0, Z99.2
Rheumatic diseases		D89.1, M05, M06.1, M30.0, M30.1, M30.8, M31.3-M31.7, M32, M33.0-M33.9, M34, M35.0, M35.2, M35.3, M45, M94.1

© 2021 Havard A et al. *JAMA Network Open*.

eTable 2. Diagnosis and Procedure Codes Used to Identify Patients With a History of Cardiovascular Disease

History of major cardiovascular diseases	ICD-10-AM Diagnosis Codes
Heart failure & cardiomyopathy	I11.0, I13.0, I13.2, I42, I43, I50, J81
Acute coronary syndrome	I20.0, I21-22
Other ischaemic heart disease	I20 (except for I20.0), I23-25
Cerebrovascular disease	I61-I67, I69, G45, G46
Peripheral arterial disease	I70.1-I70.9, I73.9, I74.2-I74.9, K55.0, K55.1
**History of cardiovascular procedures**	**Procedure[a] and ICD-10-AM Diagnosis Codes**
Percutaneous coronary interventions	35304-00, 35305-00, 35304-01, 35305-01, 38300-00, 38303-00 (block: 670) 35310-00, 35310-01, 35310-02, 35310-03, 35310-04, 35310-05, 38306-00, 38306-01, 38306-02, 38306-03, 38306-05 (block: 671) and diagnosis codes I20–I25
Coronary artery bypass grafting	38497-00 to 38497-07, 38500-00 to 38500-04, 38503-00 to 38503-04, 90201-00 to 90201-03, 38500-05, 38503-05 (blocks: 672–679) and diagnosis codes I20–I25, I34, I35

[a] Procedures recorded in the NSW APDC are coded according Australian Classification of Health Interventions

© 2021 Havard A et al. *JAMA Network Open*.

eTable 3. Baseline Characteristics of Smoking Cessation Pharmacotherapy Initiators in the Comparison of Varenicline and Bupropion, for Analyses of ACS, Stroke and Cardiovascular Death h

			Standardised difference			
**Characteristic[a]**	**Varenicline N = 342,360 n (%)**	**Bupropion N = 10,467 n (%)**	**Before weighting**	**After weighting, analysis of ACS**	**After weighting, analysis of stroke**	**After weighting, analysis of CV death**
**Age in years, mean (SD)**	43.7 (14.1)	43.6 (14.2)	0.066	0.045	0.050	0.042
**Women**	157,762 (46.1)	5,040 (48.1)	0.041	0.014	0.014	0.012
**Beneficiary category**						
General	196,481 (57.4)	5,764 (55.1)	0.047	0.037	0.037	0.033
Concessional	144,008 (42.1)	4,630 (44.2)	0.044	0.037	0.037	0.033
Veterans	1,872 (0.5)	73 (0.7)	0.019	0.000	0.000	0.002
**Socioeconomic status**						
Quintile 1 (most disadvantaged)	63,406 (18.5)	1,806 (17.3)	0.033	0.061	0.062	0.060
Quintile 2	77,125 (22.5)	2,109 (20.1)	0.058	0.006	0.009	0.009
Quintile 3	91,161 (26.6)	2,660 (25.4)	0.028	0.039	0.037	0.039
Quintile 4	65,347 (19.1)	1,912 (18.3)	0.021	0.082	0.083	0.077
Quintile 5 (least disadvantaged)	45,321 (13.2)	1,980 (18.9)	0.155	0.076	0.076	0.076
**Remoteness of residence**						
Major cities	174,040 (50.8)	5,860 (56.0)	0.103	0.017	0.020	0.019
Inner regional	86,691 (25.3)	2,432 (23.2)	0.049	0.043	0.048	0.042
Outer regional	72,672 (21.2)	1,888 (18.0)	0.080	0.009	0.007	0.012
Remote	5,426 (1.6)	228 (2.2)	0.044	0.096	0.094	0.096
Very remote	3,531 (1.0)	58 (0.6)	0.054	0.008	0.006	0.009
**Index prescription, year**						
2008	77,875 (22.7)	4,529 (43.3)	0.447	0.017	0.016	0.019
2009	76,028 (22.2)	1,561 (14.9)	0.188	0.004	0.005	0.002
2010	61,587 (18.0)	1,041 (9.9)	0.234	0.001	0.002	0.002
2011	39,762 (11.6)	721 (6.9)	0.164	0.006	0.004	0.007
2012	27,427 (8.0)	695 (6.6)	0.053	0.006	0.002	0.006
2013	22,642 (6.6)	646 (6.2)	0.018	0.011	0.013	0.012
2014	19,796 (5.8)	655 (6.3)	0.020	0.000	0.003	0.003
2015	17,243 (5.0)	619 (5.9)	0.039	0.001	0.001	0.003
**Morbidities & medicine use**						
Gastroesophageal reflux	84,805 (24.8)	2,657 (25.4)	0.014	0.028	0.029	0.025
Diabetes	20,665 (6.0)	638 (6.1)	0.003	0.002	0.005	0.001
Blood disorder	32,110 (9.4)	962 (9.2)	0.007	0.012	0.017	0.014
Arrhythmia	3,427 (1.0)	104 (1.0)	0.001	0.010	0.013	0.009
Hypertension	36,293 (10.6)	1,090 (10.4)	0.006	0.021	0.019	0.017
Hyperlipidemia	59,731 (17.4)	1,701 (16.3)	0.032	0.040	0.040	0.041
Oral corticosteroid	38,705 (11.3)	1,128 (10.8)	0.017	0.030	0.029	0.033

© 2021 Havard A et al. *JAMA Network Open*.

			Standardised difference			
**Characteristic**[a]	**Varenicline N = 342,360 n (%)**	**Bupropion N = 10,467 n (%)**	**Before weighting**	**After weighting, analysis of ACS**	**After weighting, analysis of stroke**	**After weighting, analysis of CV death**
Thyroid disease	9,192 (2.7)	339 (3.2)	0.033	0.011	0.007	0.006
Malignant neoplasms	4,477 (1.3)	144 (1.4)	0.006	0.012	0.008	0.006
NSAIDs	87,578 (25.6)	2,845 (27.2)	0.036	0.033	0.036	0.030
Epilepsy	15,814 (4.6)	697 (6.7)	0.088	0.004	0.003	0.002
Psychotic illness	17,065 (5.0)	1,067 (10.2)	0.198	0.027	0.023	0.031
Anxiety	42,253 (12.3)	1,688 (16.1)	0.109	0.027	0.025	0.023
Mood disorder	97,149 (28.4)	3,824 (36.5)	0.175	0.041	0.042	0.037
Alcohol or drug dependence	21,314 (6.2)	918 (8.8)	0.097	0.015	0.011	0.013
Chronic airway disease	92,486 (27.0)	2,879 (27.5)	0.011	0.020	0.023	0.020
Kidney disease	3,141 (0.9)	88 (0.8)	0.008	0.009	0.007	0.006
Rheumatic diseases	534 (0.2)	18 (0.2)	0.004	0.022	0.029	0.021
Heart failure & cardiomyopathy	797 (0.2)	19 (0.2)	0.011	0.004	0.005	0.002
Acute coronary syndrome	6,210 (1.8)	163 (1.6)	0.020	0.020	0.014	0.026
Other ischaemic heart disease	8,427 (2.5)	246 (2.4)	0.007	0.005	0.006	0.012
Cerebrovascular disease	3,155 (0.9)	94 (0.9)	0.002	0.004	0.001	0.009
Peripheral arterial disease	1,274 (0.4)	32 (0.3)	0.011	0.003	0.002	0.006
Percutaneous coronary interventions	1,131 (0.3)	29 (0.3)	0.010	0.003	0.004	0.000
Coronary artery bypass grafting	843 (0.2)	25 (0.2)	0.001	0.007	0.004	0.002

[a] < 0.1% of participants had missing data for these characteristics. Such participants are removed from the analysis due to the inability to compute their propensity score.

© 2021 Havard A et al. *JAMA Network Open*.

eTable 4. Baseline Characteristics of Smoking Cessation Pharmacotherapy Initiators in the Comparison of Varenicline and NRT Patch, for Analyses of ACS, Stroke and Cardiovascular Death

			Standardised difference			
**Characteristic[a]**	**Varenicline N = 123,128 n (%)**	**NRT N = 92,237 n (%)**	**Before weighting**	**After weighting, analysis of ACS**	**After weighting, analysis of stroke**	**After weighting, analysis of CV death**
**Age in years, mean (SD)**	41.9 (14.2)	49.8 (14.9)	0.548	0.002	0.003	0.003
**Women**	52,702 (42.8)	48,073 (52.1)	0.187	0.004	0.005	0.005
**Beneficiary category**						
General	73,441 (59.6)	26,074 (28.3)	0.666	0.007	0.008	0.008
Concessional	49,261 (40.0)	65,439 (70.9)	0.655	0.020	0.021	0.022
Veterans	426 (0.3)	724 (0.8)	0.058	0.075	0.075	0.077
**Socioeconomic status**						
Quintile 1 (most disadvantaged)	24,916 (20.2)	21,074 (22.8)	0.064	0.009	0.008	0.009
Quintile 2	25,785 (20.9)	20,848 (22.6)	0.040	0.017	0.019	0.018
Quintile 3	31,887 (25.9)	23,450 (25.4)	0.011	0.007	0.007	0.006
Quintile 4	23,251 (18.9)	16,479 (17.9)	0.026	0.008	0.007	0.007
Quintile 5 (least disadvantaged)	17,290 (14.0)	10,386 (11.3)	0.084	0.011	0.012	0.011
**Remoteness of residence**						
Major cities	66,313 (53.9)	48,855 (53.0)	0.018	0.018	0.019	0.019
Inner regional	28,478 (23.1)	23,844 (25.9)	0.063	0.053	0.053	0.053
Outer regional	25,651 (20.8)	17,745 (19.2)	0.040	0.033	0.032	0.033
Remote	1,547 (1.3)	1,033 (1.1)	0.013	0.008	0.008	0.006
Very remote	1,138 (0.9)	760 (0.8)	0.011	0.005	0.005	0.005
**Index prescription, year**						
2011	40,330 (32.8)	41,741 (45.3)	0.258	0.003	0.001	0.002
2012	26,805 (21.8)	18,086 (19.6)	0.053	0.000	0.000	0.000
2013	21,522 (17.5)	12,457 (13.5)	0.110	0.000	0.000	0.000
2014	18,510 (15.0)	10,202 (11.1)	0.118	0.003	0.001	0.001
2015	15,961 (13.0)	9,750 (10.6)	0.074	0.001	0.001	0.002
**Morbidities & medicine use**						
Gastroesophageal reflux	26,810 (21.8)	33,978 (36.8)	0.336	0.005	0.006	0.006
Diabetes	7,005 (5.7)	10,174 (11.0)	0.194	0.001	0.001	0.002
Blood disorder	9,318 (7.6)	16,588 (18.0)	0.316	0.013	0.012	0.013
Arrhythmia	898 (0.7)	1,938 (2.1)	0.116	0.005	0.005	0.004
Hypertension	11,466 (9.3)	18,925 (20.5)	0.319	0.008	0.008	0.008
Hyperlipidemia	17,882 (14.5)	26,392 (28.6)	0.348	0.006	0.006	0.006
Oral corticosteroid	13,637 (11.1)	19,222 (20.8)	0.269	0.010	0.009	0.009
Thyroid disease	2,999 (2.4)	4,776 (5.2)	0.144	0.002	0.002	0.002
Malignant neoplasms	1,447 (1.2)	2,342 (2.5)	0.101	0.003	0.003	0.004
NSAIDs	27,067 (22.0)	32,736 (35.5)	0.302	0.006	0.005	0.005

© 2021 Havard A et al. *JAMA Network Open*.

			Standardised difference			
**Characteristic[a]**	**Varenicline N = 123,128 n (%)**	**NRT N = 92,237 n (%)**	**Before weighting**	**After weighting, analysis of ACS**	**After weighting, analysis of stroke**	**After weighting, analysis of CV death**
Epilepsy	4,646 (3.8)	9,248 (10.0)	0.249	0.015	0.010	0.012
Psychotic illness	6,323 (5.1)	14,270 (15.5)	0.345	0.027	0.024	0.025
Anxiety	13,465 (10.9)	24,297 (26.3)	0.404	0.020	0.019	0.019
Mood disorder	32,010 (26.0)	43,894 (47.6)	0.459	0.007	0.007	0.007
Alcohol or drug dependence	7,321 (5.9)	13,179 (14.3)	0.279	0.024	0.023	0.023
Chronic airway disease	29,996 (24.4)	37,647 (40.8)	0.357	0.009	0.008	0.008
Kidney disease	918 (0.7)	1,736 (1.9)	0.100	0.010	0.009	0.010
Rheumatic diseases	109 (0.1)	292 (0.3)	0.051	0.007	0.008	0.010
Heart failure & cardiomyopathy	222 (0.2)	650 (0.7)	0.079	0.006	0.004	0.005
Acute coronary syndrome	1,602 (1.3)	3,502 (3.8)	0.159	0.024	0.023	0.023
Other ischaemic heart disease	2,042 (1.7)	4,349 (4.7)	0.175	0.014	0.013	0.015
Cerebrovascular disease	848 (0.7)	2,161 (2.3)	0.136	0.011	0.011	0.009
Peripheral arterial disease	372 (0.3)	771 (0.8)	0.071	0.005	0.003	0.005
Percutaneous coronary interventions	278 (0.2)	569 (0.6)	0.060	0.002	0.004	0.003
Coronary artery bypass grafting	208 (0.2)	480 (0.5)	0.060	0.002	0.002	0.001

[a] < 0.1% of participants had missing data for these characteristics. Such participants are removed from the analysis due to the inability to compute their propensity score.

© 2021 Havard A et al. *JAMA Network Open*.

eTable 5. Baseline Characteristics of Smoking Cessation Pharmacotherapy Initiators in the Comparison of NRT Patch and Bupropion, for Analyses of ACS, Stroke and Cardiovascular Death

			Standardised difference			
**Characteristic[a]**	**NRT N = 102,913 n (%)**	**Bupropion N = 6,084 n (%)**	**Before weighting**	**After weighting, analysis of ACS**	**After weighting, analysis of stroke**	**After weighting, analysis of CV death**
**Age in years, mean (SD)**	49.8 (14.9)	42.9 (13.6)	0.486	0.025	0.010	0.013
**Women**	53,693 (52.2)	3,043 (50.0)	0.043	0.023	0.032	0.006
**Beneficiary category**						
General	29,408 (28.6)	3,432 (56.4)	0.587	0.015	0.007	0.009
Concessional	72,711 (70.7)	2,615 (43.0)	0.582	0.009	0.031	0.025
Veterans	794 (0.8)	37 (0.6)	0.020	0.099	0.139	0.128
**Socioeconomic status**						
Quintile 1 (most disadvantaged)	23,647 (23.0)	1,080 (17.8)	0.130	0.058	0.045	0.054
Quintile 2	23,323 (22.7)	1,206 (19.8)	0.069	0.023	0.028	0.027
Quintile 3	26,365 (25.6)	1,563 (25.7)	0.001	0.015	0.000	0.008
Quintile 4	18,278 (17.8)	1,147 (18.9)	0.028	0.085	0.056	0.071
Quintile 5 (least disadvantaged)	11,301 (11.0)	1,088 (17.9)	0.197	0.031	0.041	0.034
**Remoteness of residence**						
Major cities	54,016 (52.5)	3,232 (53.1)	0.013	0.058	0.046	0.046
Inner regional	26,739 (26.0)	1,482 (24.4)	0.037	0.075	0.043	0.053
Outer regional	20,111 (19.5)	1,196 (19.7)	0.003	0.009	0.023	0.013
Remote	1,186 (1.2)	116 (1.9)	0.062	0.066	0.048	0.058
Very remote	861 (0.8)	57 (0.9)	0.011	0.013	0.014	0.021
**Index prescription, year**						
2011	42,164 (41.0)	1,305 (21.5)	0.431	0.004	0.018	0.005
2012	20,264 (19.7)	1,189 (19.5)	0.004	0.031	0.034	0.028
2013	15,008 (14.6)	1,215 (20.0)	0.143	0.012	0.003	0.009
2014	12,772 (12.4)	1,184 (19.5)	0.194	0.007	0.013	0.012
2015	12,705 (12.3)	1,190 (19.6)	0.198	0.025	0.000	0.022
**Morbidities & medicine use**						
Gastroesophageal reflux	38,297 (37.2)	1,594 (26.2)	0.238	0.006	0.013	0.013
Diabetes	11,577 (11.2)	395 (6.5)	0.168	0.010	0.009	0.003
Blood disorder	18,564 (18.0)	537 (8.8)	0.273	0.024	0.030	0.037
Arrhythmia	2,138 (2.1)	64 (1.1)	0.083	0.017	0.069	0.024
Hypertension	21,311 (20.7)	731 (12.0)	0.237	0.000	0.011	0.019
Hyperlipidemia	29,724 (28.9)	1,018 (16.7)	0.293	0.036	0.008	0.040
Oral corticosteroid	22,106 (21.5)	880 (14.5)	0.184	0.015	0.009	0.036
Thyroid disease	5,374 (5.2)	242 (4.0)	0.059	0.050	0.045	0.056
Malignant neoplasms	2,629 (2.6)	78 (1.3)	0.093	0.032	0.004	0.013
NSAIDs	37,240 (36.2)	1,622 (26.7)	0.206	0.002	0.029	0.018

© 2021 Havard A et al. *JAMA Network Open*.

			Standardised difference			
**Characteristic[a]**	**NRT N = 102,913 n (%)**	**Bupropion N = 6,084 n (%)**	**Before weighting**	**After weighting, analysis of ACS**	**After weighting, analysis of stroke**	**After weighting, analysis of CV death**
Epilepsy	10,158 (9.9)	446 (7.3)	0.090	0.021	0.036	0.030
Psychotic illness	15,561 (15.1)	748 (12.3)	0.082	0.026	0.013	0.015
Anxiety	27,001 (26.2)	1,181 (19.4)	0.163	0.014	0.022	0.011
Mood disorder	49,104 (47.7)	2,574 (42.3)	0.109	0.014	0.031	0.009
Alcohol or drug dependence	14,376 (14.0)	575 (9.4)	0.141	0.011	0.008	0.014
Chronic airway disease	42,658 (41.5)	1,780 (29.3)	0.257	0.013	0.009	0.019
Kidney disease	1,919 (1.9)	59 (1.0)	0.076	0.010	0.028	0.012
Rheumatic diseases	312 (0.3)	5 (0.1)	0.050	0.031	0.002	0.024
Heart failure & cardiomyopathy	724 (0.7)	16 (0.3)	0.063	0.003	0.020	0.023
Acute coronary syndrome	3,891 (3.8)	81 (1.3)	0.156	0.001	0.029	0.007
Other ischaemic heart disease	4,853 (4.7)	119 (2.0)	0.154	0.001	0.011	0.012
Cerebrovascular disease	2,424 (2.4)	53 (0.9)	0.118	0.088	0.064	0.078
Peripheral arterial disease	877 (0.9)	25 (0.4)	0.056	0.016	0.031	0.085
Percutaneous coronary interventions	635 (0.6)	18 (0.3)	0.048	0.033	0.027	0.030
Coronary artery bypass grafting	531 (0.5)	13 (0.2)	0.050	0.027	0.031	0.021

[a] < 0.1% of participants had missing data for these characteristics. Such participants are removed from the analysis due to the inability to compute their propensity score.

© 2021 Havard A et al. *JAMA Network Open*.

eTable 6. Hazard Ratios for Cardiovascular Outcomes Associated With Smoking Cessation Pharmacotherapy Initiation, for Each Pairwise Comparison[a]

	Exposure	Number of individuals[b]	Number of events	Person-years	Incidence rate, per 1000 person years (95% CI)	Hazard ratio (95% CI)
MACE	Varenicline	333,074	1338	164,197	8.15 (7.73, 8.60)	0.81 (0.55, 1.20)
	Bupropion	10,159	50	4,974	10.03 (6.87, 15.25)	1.00 (ref)
ACS	Varenicline	334,074	1001	164,197	6.10 (5.73, 6.49)	0.73 (0.48, 1.10)
	Bupropion	10,159	42	4,975	8.38 (5.64, 13.03)	1.00 (ref)
Stroke	Varenicline	334,074	243	164,197	1.48 (1.31, 1.68)	[d]
	Bupropion	10,158	<5	[c]	0.63 (0.14, 6.02)	
CV death	Varenicline	334,074	195	164,199	1.19 (1.04,1.37)	0.94 (0.33, 2.64)
	Bupropion	10,158	6	4,976	1.27 (0.41, 5.73)	1.00 (ref)
MACE	Varenicline	115,460	578	57,108	10.13 (8.93, 11.53)	0.93 (0.79, 1.08)
	NRT	87,053	462	42,204	10.94 (10.06, 11.92)	1.00 (ref)
ACS	Varenicline	115,457	417	57,085	7.30 (6.33, 847)	0.96 (0.81, 1.15)
	NRT	87,053	319	42,205	7.57 (6.83, 841)	1.00 (ref)
Stroke	Varenicline	115,451	105	57,068	1.85 (1.40, 2.48)	0.83 (0.59, 1.16)
	NRT	87,053	94	42,207	2.23 (1.87, 2.69)	1.00 (ref)
CV death	Varenicline	115,448	86	57,069	1.51 (1.12, 2.10)	0.67 (0.47, 0.95)
	NRT	97,053	96	42,201	2.26 (1.93, 2.68)	1.00 (ref)
MACE	NRT	96,165	695	47,026	14.78 (13.74, 15.91)	1.11 (0.64, 1.93)
	Bupropion	5,471	34	2,519	13.33 (7.84, 24.59)	1.00 (ref)
ACS	NRT	96,165	455	47,026	9.68 (8.84, 10.61)	0.57 (0.30, 1.08)
	Bupropion	5,470	43	2,524	17.12 (9.06,36.44)	1.00 (ref)
Stroke	NRT	96,165	154	47,027	3.28 (2.81, 3.85)	[d]
	Bupropion	5,473	<5	[c]	0.69 (0.09, 17.41)	
CV death	NRT	96,165	167	47,028	3.55 (3.07, 4.13)	[d]
	Bupropion	5,474	<5	[c]	1.44 (0.37, 9.64)	

[a] Sensitivity analyses ie using an ITT approach. Treatment groups were weighted using inverse probability of treatment weighting with high dimensional propensity scores. [b]Varies across comparisons due to removal of individuals with weights ≥ 10. [c]Cell value suppressed because it was based on <5 individuals. [d] HR not reported due to there being <5 events in at least one of the exposure groups.

© 2021 Havard A et al. *JAMA Network Open*.

eTable 7. Hazard Ratios for MACE Associated With Smoking Cessation Pharmacotherapy Initiation, Among Patients With a History of Cardiovascular Disease[a]

Exposure	Number of individuals	Number of events	Person-years	Incidence rate, per 1000 person years (95% CI)	Hazard ratio (95% CI)
Varenicline	13,724	Even after weighting and trimming of weights ≥ 10, meaningful differences between groups remained. Outcomes were not examined.			
Bupropion	405				
Varenicline	3,554	70	936	74.60 (53.76, 106.61)	0.77 (0.54, 1.12)
NRT	7,812	193	1,965	98.35 (85.42, 113.82)	1.00 (ref)
NRT	8,735	Even after weighting and trimming of weights ≥ 10, meaningful differences between groups remained. Outcomes were not examined.			
Bupropion	205				

[a] Using an as-treated approach. Treatment groups were weighted using inverse probability of treatment weighting with high dimensional propensity scores.

© 2021 Havard A et al. *JAMA Network Open*.

eTable 8. Hazard Ratios for All-Cause Death Outcome Associated With Smoking Cessation Pharmacotherapy Initiation, for Each Pairwise Comparison[a]

Exposure	Number of individuals	Number of events	Person-years	Incidence rate, per 1000 person years (95% CI)	Hazard ratio (95% CI)
Varenicline	342,064	307	87,883	3.49 (3.13, 3.91)	0.43 (0.24, 0.76)
Bupropion	10,458	21	2,581	8.15 (4.69, 15.48)	1.00 (ref)
Varenicline	122,927	141	32,345	4.37 (3.35, 5.81)	0.31 (0.23, 0.41)
NRT	92,148	290	20,844	13.93 (12.72, 15.29)	1.00 (ref)
NRT	102,817	519	24414	21.28 (19.57, 23.17)	2.39 (1.03, 5.52)
Bupropion	6,052	13	1439	9.19 (4.01, 25.92)	1.00 (ref)

[a] Using an as-treated approach. Treatment groups were weighted using inverse probability of treatment weighting with high dimensional propensity scores

© 2021 Havard A et al. *JAMA Network Open*.

eFigure. High-Dimensional Propensity Score Distribution Among Smoking Cessation Pharmacotherapy Initiators Before and After Weighting

a. **Varenicline vs bupropion**

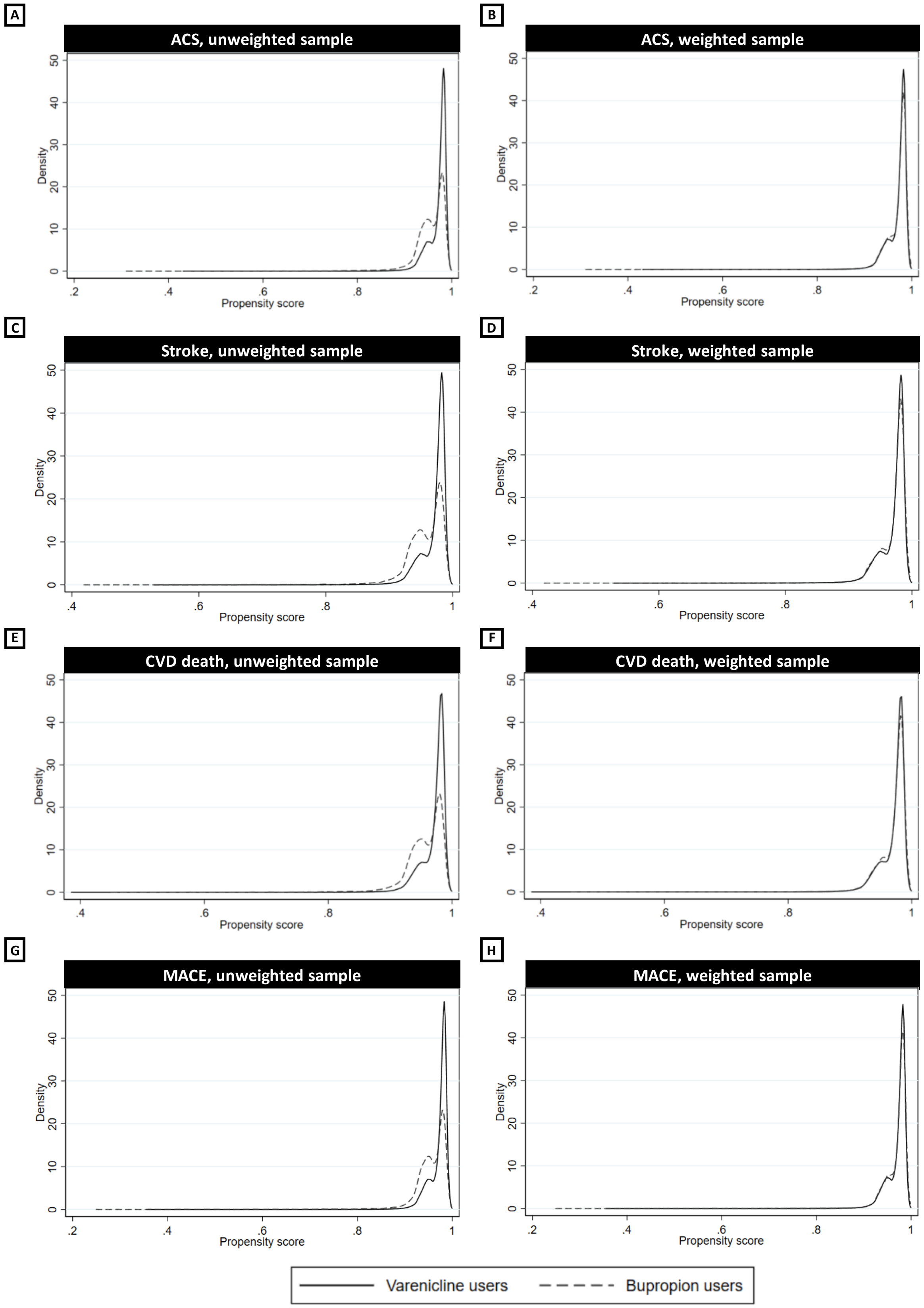

© 2021 Havard A et al. *JAMA Network Open*.

b. Varenicline vs NRT patches

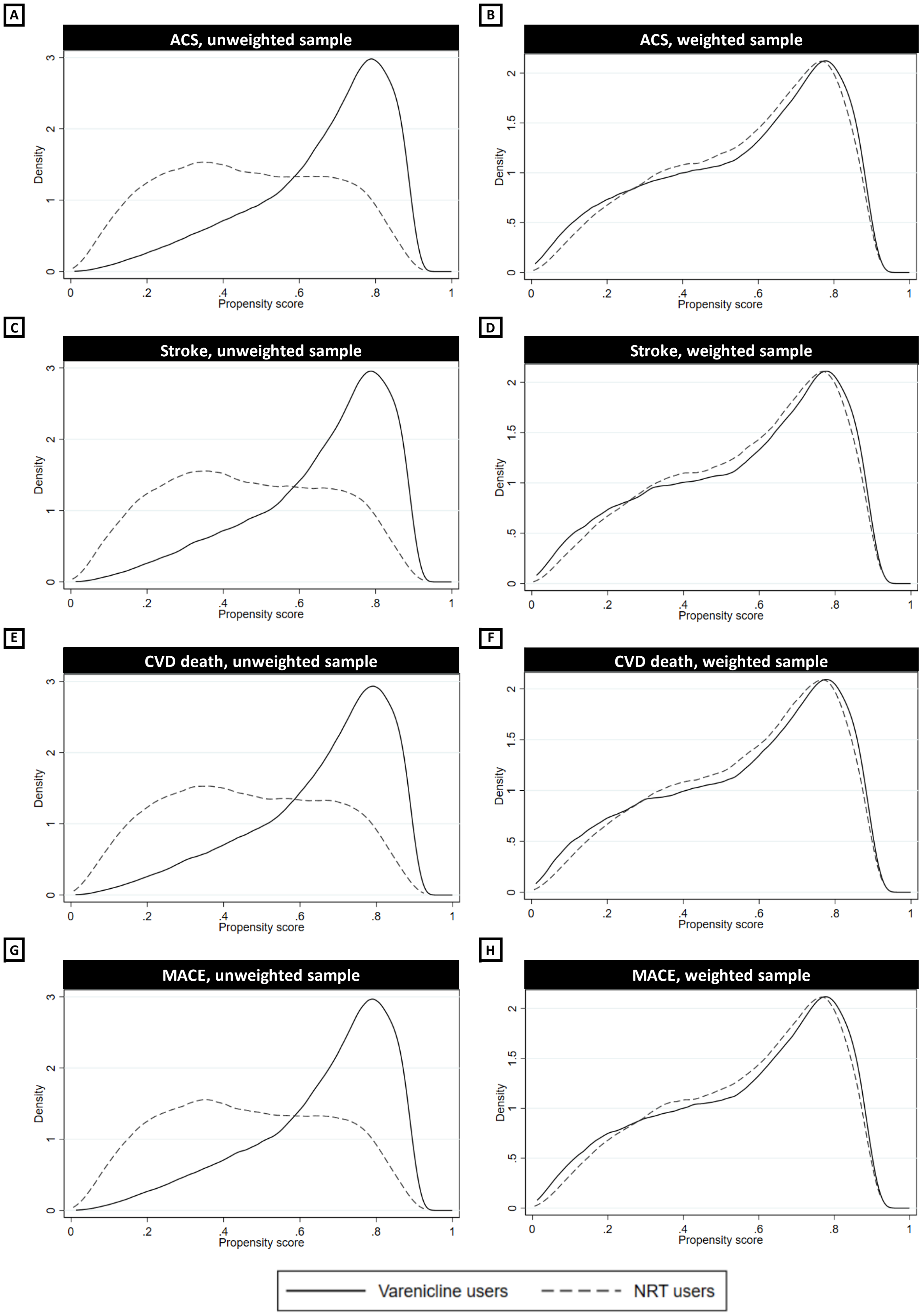

© 2021 Havard A et al. *JAMA Network Open*.

c. NRT patches vs bupropion

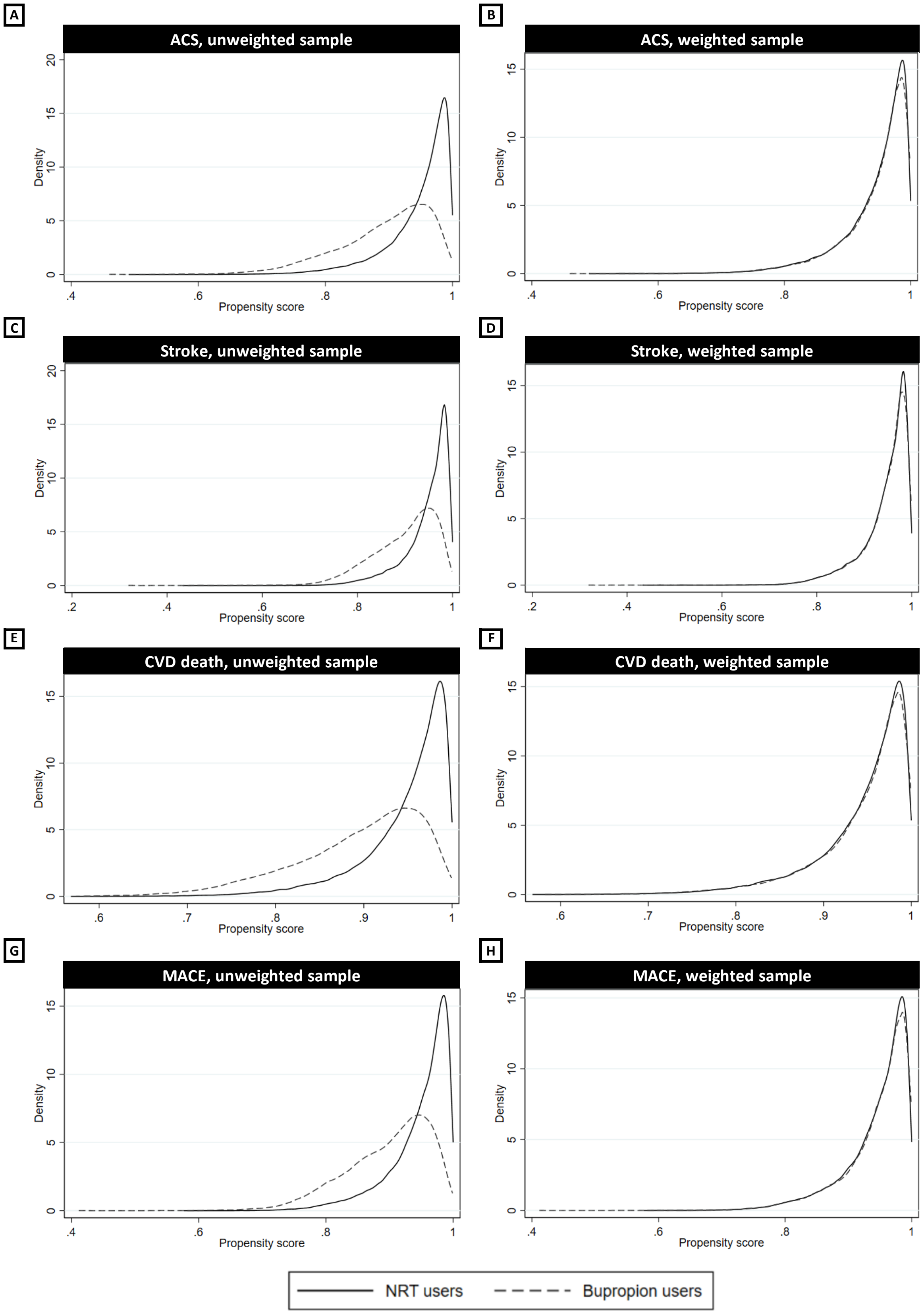

© 2021 Havard A et al. *JAMA Network Open*.
